# Novel Neuroimaging Biomarker for Sleep Quality in Insomnia Disorder: A Hypothalamus Resting State Study

**DOI:** 10.3389/fnins.2021.634984

**Published:** 2021-02-26

**Authors:** Shuang Ding, Lijuan Gao, Hanjiaerbieke Kukun, Kai Ai, Wei Zhao, Chao Xie, Yunling Wang

**Affiliations:** ^1^Department of Radiology, The First Affiliated Hospital, Xinjiang Medical University, Ürümqi, China; ^2^Philips Healthcare, Xi’an, China; ^3^Department of Radiology, The Seventh Affiliated Hospital, Xinjiang Medical University, Ürümqi, China

**Keywords:** insomnia disorder, hypothalamus, resting state functional connectivity, medial prefrontal cortex, pittsburgh sleep quality index

## Abstract

Despite striking progress in the understanding of the neurobiology of insomnia disorder (ID), about 40% of ID patients do not reach sustained remission with the primary treatments. It is necessary to reveal novel neuroimaging biomarkers for sleep quality in ID. The hypothalamus has a central role in sleep-wake regulation by communicating with different brain regions. However, the functional implications of hypothalamus circuitry with other brain areas remains largely unknown in ID. It may be speculated that dysfunctional circuitry in the hypothalamus is involved in the pathogenesis of ID. Thus, we investigated the different network organizations of the bilateral hypothalamus during the resting-state between 26 ID patients and 28 healthy controls (HC). Correlation analysis has been carried out to link the neuroimaging findings and Pittsburgh sleep quality index (PSQI) scores. Group comparisons reveal that the resting-state functional connectivity (RSFC) between the left hypothalamic region and a few other brain regions, including the medial prefrontal cortex (mPFC) and pallidum, are significantly higher in ID compared with HC. The right inferior temporal cortex showed reduced RSFC with the left hypothalamus. No significantly different RSFC between ID and HC was detected for the right hypothalamus. Positive correlations with PSQI scores were observed for RSFC strength between the left hypothalamus and bilateral mPFC (left: *r* = 0.2985, *p* = 0.0393; right: *r* = 0.3723, *p* = 0.0056). Similarly, the RSFC strength between the right hypothalamus and bilateral mPFC (left: *r* = 0.3980, *p* = 0.0029; right: *r* = 0.2972, *p* = 0.0291) also showed significant positive correlations with PSQI scores. In conclusion, we reveal a novel neuroimaging biomarker for sleep quality, i.e., the RSFC strength of the hypothalamus-mPFC pathway. Consistent with the hyperarousal model of ID, our results shed new insights into the implications of the hyper-connection within hypothalamus circuits in the pathology of the ID.

## Introduction

As the most common sleep disorder, insomnia disorder (ID) is characterized by difficulties in falling asleep, maintaining sleep, or non-restorative sleep (Riemann et al., 2015). Additionally, ID is commonly accompanied by significantly impaired daytime functioning in the absence of a specific physical, mental, or substance-related cause (Riemann et al., 2015; Min et al., 2018). Worldwide studies have demonstrated that up to 50% of the world’s population suffers from insomnia, and the prevalence of ID ranges from 3 to 5% in the general population (Riemann et al., 2010). Moreover, the prevalence of ID is still rising in younger patients. ID not only affects daily life, but also leads to functional impairment or disease, such as memory loss (Backhaus et al., 2006) and mental disorders (Riemann and Voderholzer, 2003). Despite striking progress in the understanding of the neurobiology of ID, about 40% of ID patients do not reach sustained remission with primary treatments (Morin et al., 2009). Therefore, in order to develop more effective treatment, it is necessary to discover novel neuroimaging biomarkers for sleep quality in IDs.

The pathophysiology of ID had been investigated by examining both peripheral and central nervous measurements from the perspective of the hyperarousal model (Riemann et al., 2010, 2015). Dysregulation or hyperactivity of the Hypothalamus-Pituitary-Adrenal (HPA) axis and the gonadotropin-releasing hormone have been characterized in ID (Lan et al., 2013), which had been linked to insomniac hyper-arousal (Roth et al., 2007). The hypothalamus has a central role in sleep-wake regulation by communicating with different brain regions (Saper et al., 2005). Orexinergic neurons in the hypothalamus modulate sleep-wake regulation by reinforcement of arousal pathways in the brainstem and have a direct excitatory input into the cerebral cortex and basal forebrain (Saper et al., 2010). The altered dopaminergic transmission in the hypothalamus (i.e., Dopamine D3 receptor availability) have been established to probably contribute to the development of sleep disorders in Parkinson patients (Chaudhuri et al., 2016). Similarly, in the neuroimage studies of ID, patients show a slightly decreased relative metabolism of the hypothalamus from waking to non-rapid eye movement (NREM) sleep states compared with healthy controls (HC) (Nofzinger et al., 2004). Therefore, it may be speculated that dysfunctional circuitry in the hypothalamus is involved in the pathogenesis of ID. But in fact, the hypothalamic circuits have been identified in rodent studies to reveal rich connections from and to the hypothalamus especially projections of the cortical regions and limbic system (Kampe et al., 2009).

Previous neuroimaging studies have revealed that a few brain areas may be involved in ID, such as the hippocampus, amygdala, thalamus, caudate, anterior cingulate cortex (ACC), and frontal cortex (Drummond et al., 2004; O’Byrne et al., 2014; Khazaie et al., 2017). Higher regional homogeneity (ReHo) in the insula, ACC, precentral gyrus, and fusiform gyrus had been observed in IDs (Wang et al., 2016). Similarly, increased amplitude of low-frequency fluctuation (ALFF) in the temporal and occipital lobes were also detected in IDs (Dai et al., 2014). Enhanced resting-state functional connectivity (RSFC) of the thalamus-medial prefrontal cortex (mPFC) (Min et al., 2018) and nucleus accumbens (NAc)-mPFC (Ziqiang et al., 2020) were found in IDs. With regard to abnormalities of functional networks in IDs, increased synchronicity of the bilateral anterior insula with salience network (SN) (Chen et al., 2014) and increased functional connectivity between the bilateral superior parietal lobule and several default mode network (DMN) areas (Li et al., 2014). Moreover, increased RSFC between the retrosplenial cortex and hippocampus during waking, as well as several nodes of the DMN, is associated with impaired sleep quality, such as lower sleep efficiency, lower amounts of REM sleep, and greater sleep-onset latency (Regen et al., 2016). All the findings mentioned above reveal functional abnormalities of ID, which are partially in accordance with the classic hyperarousal hypothesis for the pathology of ID. Furthermore, hypothalamic RSFC has been found to predict a pattern of regional sleep-wake cycle changes in humans, which have extended the hypothalamic circuit in the sleep of experimental animals as well as humans and enhanced our understanding of human sleep-wake regulation (Boes et al., 2018). However, the functional implications of hypothalamus circuitry with these brain areas remains largely unknown in ID.

Therefore, in the current study, we have aimed to compare RSFC organizations of the bilateral hypothalamus between ID and HC. Additionally, correlation analysis would be carried out to link the neuroimaging findings and subjective ratings of sleep quality (PSQI scores). Our research could shed new insights on the hyperarousal model of ID and reveal novel neuroimaging biomarkers for sleep quality in ID.

## Materials and Methods

Informed written consent was obtained from all participants. The protocol was approved by the local Ethics Committee.

### Participants

According to DSM-IV-TR criteria, twenty-six subjects diagnosed with ID and 28 healthy controls (HC) without sleep complaints were recruited through local advertisements (Table 1). They were matched for age, sex, and education. All patients complained of difficulty in falling asleep, maintaining sleep, or early awakening (lasted for at least 1 month), which were not secondary to any medications, substance abuse, or physical or psychiatric disorders. They did not suffer from any other psychiatric disorders or sleep disorders including hypersomnia, parasomnia, circadian rhythm sleep disorder, sleep-related movement disorder, or sleep-related breathing disorder. The following criteria were used to screen healthy controls: (a) they have no sleep complaints or psychiatric disorders; (b) the total Pittsburgh sleep quality index score (PSQI) < 5; and (c) they have no history of psychiatric or neurologic diseases. Exclusion criteria included (1) sleep problems caused by organic disease or severe mental disease; (2) a history of neurological or other physical diseases, such as cardiac, hepatic, renal, endocrine, and respiratory diseases; (3) any substance use disorder; (4) any medication that might affect sleep or cerebral function taken within 1 month before the scans; (5) women who were pregnant, nursing, or menstruating. Prior to the MRI scanning, the PSQI was employed to assess sleep quality (Buysse et al., 1989). Meanwhile, the Self-Rating Anxiety Scale (SAS) (Zung, 1971) and Self-Rating Depression Scale (SDS) (Zung, 1965) were used for the assessment of depression and anxiety.

**TABLE 1:** Demographic information of the participants in current study.

**Variable**	**Control (*n* = 28)**	**Insomnia (*n* = 26)**	***t*/χ^2^-value**	***p*-value**
Age (years)	39.96 ± 9.13	41.50 ± 9.01	0.622	0.537
Gender (male/female)^*a*^	13/15	8/18	1.391	0.238
Education (years)	13.64 ± 2.97	9.65 ± 3.73	4.325	0.00
PSQI	4.29 ± 2.05	13.62 ± 3.56	11.691	0.00
SAS	29.75 ± 7.41	53.04 ± 10.12	9.701	0.00
SDS	17.14 ± 11.15	46.00 ± 9.61	10.151	0.00

*^*a*^Chi-square test.*

### MRI Data Acquisitions

Whole-brain fMRI data were obtained using a 3.0 T scanner (Philips Achieva 3.0T, Best, Netherlands) at the Department of Radiology of the Seventh Affiliated Hospital, Xinjiang Medical University. The high-resolution T1 weighted brain anatomical images (scanning parameters: TR = 8.1 ms; TE = 3.7 ms; Flip Angle = 7 degrees; Resolution = 1mm × 1mm × 1 mm; Acquisition matrix = 256 × 256; FOV = 256 mm × 256 mm; slice thickness = 1 mm; slice number = 162) were obtained. Functional data were collected using echo-planar imaging sequences. All subjects were instructed to keep their eyes closed and not to think about anything particular during the resting state MR acquisition. For BOLD images collection, the following sequence was used: TR = 2,000 ms, TE = 30 ms, FOV = 256 mm^2^, matrix 64 × 64, flip angle 90°, voxel size 3.5 mm × 3.5 mm × 3.5 mm, slice thickness 3.5 mm, and the images were acquired in an interleaved order without gaps. Each brain volume comprised 43 axial slices and each functional run contained 215 volumes, resulting in a total scan time of 7 min 10 s.

### Resting-State fMRI Data Processing

Resting-state data was analyzed using DPABI: Data Processing and Analysis for (Resting-State) Brain Imaging (Yan et al., 2016), which is based on statistical parametric mapping (SPM8)^1^. The first five volumes were removed to allow for scanner calibration and participants’ adaptation to the scanning environment. The remaining functional images were motion corrected and co-registered to the 3D-T1 image. The structural image was then normalized to the Montreal Neurological Institute template. The derived spatial transformation parameters were used to normalize the functional images with a resampling voxel size of 3 mm × 3 mm × 3 mm. Finally, the normalized functional images were smoothed with a three-dimensional isotropic Gaussian kernel, full-width at half-maximum (FWHM) = 8 mm. To reduce low-frequency drifts and high frequency physiological noise, a temporal filter (0.01–0.08 Hz) was applied to the smoothed functional images. Additionally, nuisance regression was performed by including the global signal, white matter, cerebrospinal fluid (CSF), and the six-head motion parameters as covariates. No participant had head motion with more than 2.0 mm maximum displacement or 2.0° of any angular motion. Regions of interest (ROIs) in the bilateral hypothalamus (Index 363, 364) were defined according to Talairach.nii^2^, see Supplementary Figure 1 (Lancaster et al., 2000). RSFC was examined based on the seed-based correlation approach. For each ROI (left and right hypothalamus), the resting-state fMRI time series was extracted from preprocessed data by averaging the BOLD signals of all voxels within each ROI. RSFC analysis was conducted between the seeding reference and the rest of the whole brain in a voxel-wise manner. The resulting *r*-value maps were subsequently transformed to approximate Gaussian distribution using Fisher’s *z* transformation.

### Statistical Analysis

For between-group comparison, two-sample *t*-tests were used to compare *z*-value maps between ID and HC (family-wise error correction at *p* < 0.05). To investigate the association between the RSFC findings of these regions and severity of insomnia, Pearson correlation was employed to calculate the correlation coefficients between the abnormal *z*-values and PSQI scores in all participants.

## Results

Detailed demographic information can be found in Table 1. There were no significant differences in age or gender between ID patients and HCs. Impaired sleep quality measured by PSQI scores was found in ID patients (13.62 ± 3.56) compared with HCs (4.29 ± 2.05). Higher SAS (*t* = 9.701, *p* < 0.001) and SDS (*t* = 10.15, *p* < 0.001) were also detected in ID patients.

**FIGURE 1:**
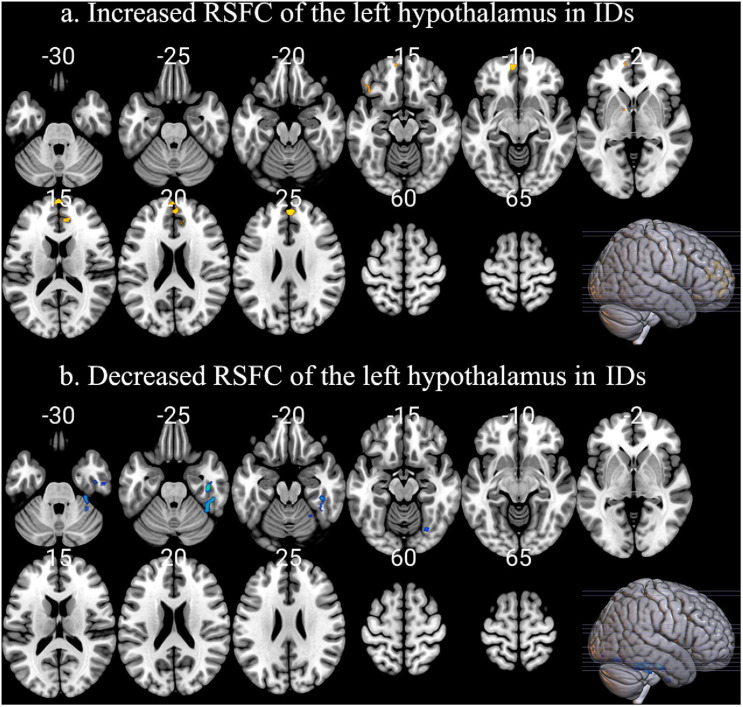
The abnormal RSFC patterns of left hypothalamus in IDs. **(a)** Compared with HC, ID had significantly increased RSFC between the left hypothalamic and a series of brain regions, including the bilateral medial prefrontal cortex (mPFC) and left pallidum. **(b)** The right inferior temporal cortex showed reduced RSFC with the left hypothalamus.

**FIGURE 2:**
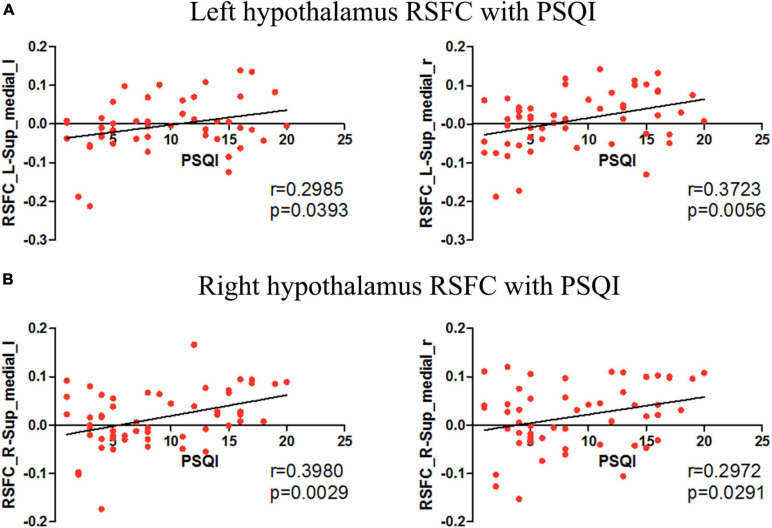
The correlation between the hypothalamus RSFC and PSQI. **(A)** Correlations were observed between the left hypothalamus-bilateral mPFC RSFC and PSQI scores. **(B)** Correlations were observed between the right hypothalamus-bilateral mPFC RSFC and PSQI scores.

Insomnia disorder and HC showed similar positive RSFC between the bilateral hypothalamus and voxels located within or adjacent to the hypothalamus. For both groups, the hypothalamus showed significant correlation with the bilateral thalamus, striatum (caudate, putamen, nucleus accumbens, and pallidum), amygdala, hippocampus, and brain stem (pons and midbrain). Compared with HC, ID had significantly increased RSFC between the left hypothalamic and a number of brain regions, including the bilateral medial prefrontal cortex (mPFC) and left pallidum (Figure 1a). The right inferior temporal cortex showed reduced RSFC with the left hypothalamus (Figure 1b). No significant between-group difference of RSFC was detected for the right hypothalamus. Positive correlations with PSQI scores were observed for RSFC strength between the left hypothalamus and bilateral mPFC (left: *r* = 0.2985, *p* = 0.0393; right: *r* = 0.3723, *p* = 0.0056). Similarly, the RSFC strength between the right hypothalamus and bilateral mPFC (left: *r* = 0.3980, *p* = 0.0029; right: *r* = 0.2972, *p* = 0.0291) also showed significant correlations with PSQI scores (Figure 2). No other significant correlations between neuroimaging findings and PSQI were observed in the current study.

## Discussion

During the last decade, the important role of the hypothalamus in the mechanisms of sleep-wake regulation has been observed in animal studies, which were closely associated with the orexin- and melanin-concentrating hormone neurons (Saper et al., 2005). In detail, orexin neurons played crucial roles for maintenance of wakefulness and melanin-concentrating hormone (MCH)-producing neurons were important for promoting sleep (Konadhode et al., 2013; Scammell et al., 2017). For human beings, the damage in anterior parts of the hypothalamus would induce an insomnia-like phenomenon in people (Ono and Yamanaka, 2017). In parallel, the damage in the posterior parts of the hypothalamus resulted in abnormally increased sleep periods in patients (Economo, 1930). ID patients showed abnormal sleep-wake transitions, which were probably regulated by a coordinated interaction between specific neurons in the hypothalamus and brainstem (Scammell et al., 2017). Converging evidence has revealed the key brain regions that regulate sleep and arousal, including the hypothalamus, thalamus, basal ganglia, and cerebral cortex (Scammell et al., 2017). Although previous studies have observed that hypothalamic RSFC could predict a pattern of regional sleep-wake changes in humans and enhance our understanding of human sleep-wake regulation (Boes et al., 2018), they have failed to investigate the implications of the hypothalamus circuitry in the pathology of ID. To fill this gap, we have revealed the increased RSFC of the left hypothalamus with mPFC and pallidum in ID. Moreover, the RSFC strength between the hypothalamus and mPFC are correlated with PSQI scores in all participants, which could be the novel neuroimaging biomarkers for sleep quality.

In ID and HC, we identified a similar temporally correlated network of the hypothalamus throughout the brain including the thalamus, striatum, amygdala, hippocampus, and brain stem, which are involved in wake maintenance and sleep-promoting (Kaufmann et al., 2006). It is worthy to note that the hypothalamus RSFC pattern was partially overlapped with the indirect pathway of the ascending reticular activating systems (ARAS), which sends projections from the brainstem and posterior hypothalamus throughout the forebrain (Saper et al., 2001). The hypothalamus has been proved to regulate ARAS during sleep, and thus dysfunction of the hypothalamus is involved in the pathogenesis of ID. In IDs, the hypothalamus and mPFC showed smaller declines in relative glucose metabolism of hypothalamus from wakefulness to NREM sleep (Nofzinger et al., 2004). The synchronously smaller decline in the hypothalamus and mPFC indicate that they probably participate in sleep-wake transition together. Our results provide further scientific evidence for the implications of the hypothalamus circuits in ID by showing increased RSFC between the left hypothalamus and mPFC. Moreover, the RSFC strength between the left hypothalamus and bilateral mPFC are positively correlated with PSQI scores.

Additionally, increased RSFC between the left hypothalamus and the pallidum were also detected in ID. Recently, more and more attention has been paid to investigate how the basal ganglia regulate sleep-wake transition (Lazarus et al., 2012). Compared with the waking state, decreased activity in the basal ganglia has been detected during slow-wave sleep (Kaufmann et al., 2006). Here, we detected increased RSFC between the left hypothalamus and pallidum in ID (Figure 1a). The implications of the pallidum in the control of sleep and wakefulness have also been confirmed based on electrophysiology, neurotoxic lesioning, and the use of transgenic animal studies (Lazarus et al., 2012). More importantly, the activity of the pallidum and the hypothalamus is probably the major source of cortical arousal, which might explain the increased RSFC of the hypothalamus-pallidum in ID. These findings were partially consistent with the hyperarousal model of the pathophysiology of ID, which were mainly associated with overactivity of the sleep-wake transition-related regions (Nofzinger et al., 2004; Riemann et al., 2010; Kay and Buysse, 2017). In detail, wakefulness depends on orexinergic neurons activating the thalamus and the cerebral cortex, and the hypothalamus has the capacity to shut off this arousal system during sleep. The pathology of ID was closely associated with the imbalance between sleep-promoting areas in the brain (i.e., the VLPO: ventrolateral preoptic nucleus; neurotransmitter: GABA) and arousal-promoting neurons (among others orexin neurons in the lateral hypothalamus) with relative overactivity of the orexin system or a hypofunction of the VLPO. The dysfunction of the hypothalamus in the current study was evidenced by the abnormal RSFC patterns with mPFC, which can provide novel insights into the pathogenesis of ID. We have extended the previous findings of the hypothalamus in ID from regional implications into brain circuits implications by demonstrating the hyper-RSFC of the regions in sleep-wake regulation and sleep-inducing systems.

Meanwhile, the reduced RSFC between the left hypothalamus and inferior temporal cortex was detected in IDs (Figure 1b). The cortical thinning in the inferior temporal cortex has been observed in Parkinson’s disease with polysomnography-confirmed rapid eye movement (REM) sleep behavior disorder (Shady et al., 2019), which highlights the potential role of the inferior temporal cortex in sleep regulation. In addition, the inferior temporal cortex has been associated with memory (Miller et al., 1991). By acting as adaptive mnemonic filters, the inferior temporal cortex preferentially passes novel and unexpected information, which would be crucial for memory storage. Recent studies have found memory formation deficits during sleep in IDs (Nofzinger et al., 2004; Backhaus et al., 2006). Disrupted RSFC between the inferior temporal cortex and hippocampus have also been detected in patients with obstructive sleep apnea (Song et al., 2018). We suggest that the reduced RSFC between the left hypothalamus and inferior temporal cortex was probably correlated with memory deficits in IDs. Evidently, this hypothesis should be verified by including memory tasks in the future.

### Limitations

First, the cross-sectional design of the current study has failed to ascertain a causal link in the relationship between ID and disrupted RSFC of the hypothalamus, and longitudinal studies may help to resolve this question. Second, objective measures together with PSQI, such as polysomnography, should be used to assess sleep quality. Third, the findings of this study need to be verified by studies with larger sample sizes. In the current study, only the left hypothalamus has shown abnormal RSFC patterns between IDs and controls. However, the RSFC strength of the bilateral hypothalamus-mPFC correlates with PSQI. The predominant left lateralization of the findings needs to be verified by a larger sample in the future. Last but not least, neurocognitive tests can be applied to investigate more comprehensive clinical implications of the abnormal hypothalamus RSFC in ID. DSM-IV was used for the diagnosis of ID patients in the current study, and the diagnostic criteria are different from the latest version (DSM-V). This should be taken into consideration for the extension of our findings.

## Conclusion

We have revealed a novel neuroimaging biomarker for sleep quality in ID, i.e., the RSFC strength of the hypothalamus-mPFC pathway. The results of this study show partial consistency with the existing hyperarousal model of ID. It is hoped that our results may shed novel insights into the underlying mechanisms of the pathology of the ID.

## Data Availability Statement

The original contributions presented in the study are included in the article/Supplementary Material, further inquiries can be directed to the corresponding author/s.

## Ethics Statement

The studies involving human participants were reviewed and approved by informed written consent was obtained from all the participants. The protocol was approved by The First and Seventh Affiliated Hospital of Xinjiang Medical University Ethics Committee. The patients/participants provided their written informed consent to participate in this study.

## Author Contributions

SD wrote the first version of this manuscript. LG, HK, and KA carried out the data processing and statistical analysis. WZ and CX were responsible for the data collection. YW contributed to the conception and design of this manuscript. All authors contributed to the manuscript revision.

## Conflict of Interest

KA was employed by company Philips Healthcare, China. The remaining authors declare that the research was conducted in the absence of any commercial or financial relationships that could be construed as a potential conflict of interest.

## Footnotes

1 http://www.fil.ion.ucl.ac.uk/spm

2 http://talairach.org/
