# Supplementary figures and images for: Novel Neuroimaging Biomarker for Sleep Quality in Insomnia Disorder: A Hypothalamus Resting State Study
Source: Front Neurosci. 2021 Feb 26;15:634984. doi: 10.3389/fnins.2021.634984 (PMC7953135; doi:10.3389/fnins.2021.634984)

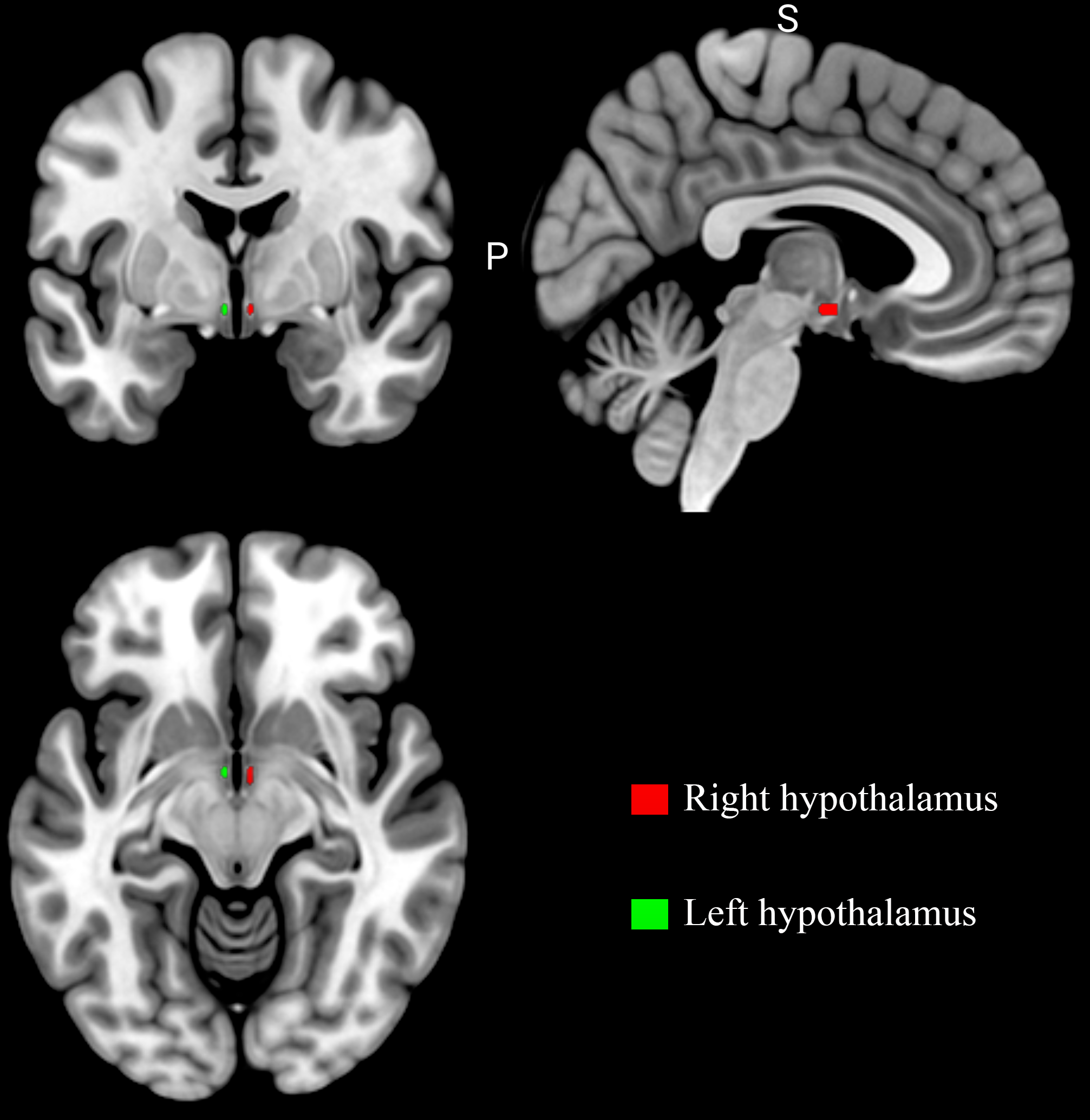

Supplement: Supplementary file 1 [file Image_1.TIF]
